# 

**Published:** 1885-09

**Authors:** 


					﻿OLD SERIES
Vol. xxv
1855 O
/ J /
new series
Vol. II.—No. 7.


^Wby1
W.F. WESTMORELAND. M®
H.V.M.MILLER.M.D.LL.D.W
JAMES.A.GRAY. M.D.
published By james.p.harrison&cqS
tflTLANTA ,GA.
SUBSCRIPTION: $2.50 A YEAR.
				

